# Acute Intracerebral Hemorrhage Associated with Extensive Venous Thrombosis Due to Spontaneous Heparin-Induced Thrombocytopenia After Total Knee Replacement: A Case Report

**DOI:** 10.3390/hematolrep17020012

**Published:** 2025-03-13

**Authors:** Mehdi Kashani, Meghan Brown, Juan Pablo Domecq Graces

**Affiliations:** Department of Nephrology, Hypertension, and Critical Care Mayo Clinic, 200 First St. SW, Rochester, MN 55905, USAdomecq.juan@mayo.edu (J.P.D.G.)

**Keywords:** spontaneous heparin-induced thrombocytopenia (S-HIT), cerebral venous sinus thrombosis (CVST), intracerebral hemorrhage, heparin-induced thrombocytopenia (HIT), platelet factor 4 (PF4) antibodies, total knee replacement, venous thrombosis, pulmonary embolism, bivalirudin anticoagulation, autoimmune thrombocytopenia

## Abstract

Introduction: Heparin-induced thrombocytopenia (HIT) is an autoimmune life-threatening prothrombotic syndrome associated with low platelet count after heparin exposure. Spontaneous heparin-induced thrombocytopenia (S-HIT) is an even less frequent variant of HIT, with only a handful of reports available in the literature, where unexplained thrombocytopenia and/or thrombosis without recent heparin exposure occurs in the setting of positive anti-PF4 antibodies. Case Presentation: We report a case of S-HIT associated with pulmonary artery embolism, left internal jugular vein, and cerebral vein sinus thrombosis complicated with ipsilateral acute intracerebral hemorrhage. Discussion: It is important to highlight that in patients with otherwise unexplained thrombocytopenia and prior exposure to an inflammatory process, S-HIT should be on the differential. Conclusions: Recognition and avoidance of heparin exposure is the most important aspect of S-HIT, as the management is otherwise similar to HIT.

## 1. Introduction

Heparin-induced thrombocytopenia (HIT) is an immune prothrombotic syndrome that manifests with arterial and/or venous thrombosis episodes associated with unexplained moderate to severe thrombocytopenia. HIT is rare but life-threatening. There is a production of antibodies directed against endogenous platelet factor 4 (PF4) in complex with heparin [1,2]. These antibodies bind and activate cellular Fc-γRIIa on platelets resulting in thrombosis. Other complications depend on the location and severity of the thrombotic events. The overall mortality rate could go as high as 20% [3].

Spontaneous HIT (S-HIT) is an even less frequent variant of HIT, with only a handful of reports available in the literature, where unexplained thrombocytopenia and/or thrombosis without recent heparin exposure occurs and anti-PF4 antibodies of the immunoglobulin G (IgG) subclass are strongly positive [4]. It is not surprising that S-HIT diagnosis is particularly challenging, where a high level of suspicion is needed to avoid a delayed diagnosis. Thrombosis occurs in up to 50% of individuals with HIT, and S-HIT shows a similar risk [4]. In previously published reports, S-HIT was associated with pulmonary artery embolisms, bilateral adrenal gland necrosis, lower extremity deep vein thrombosis, and vertebral artery thrombosis with secondary stroke [5,6,7,8].

Cerebral venous sinus (CVS) Thrombosis is also a rare but life-threatening entity that accounts for 1% of all strokes and is associated with a 10% mortality rate [1]. CVS thrombosis has been described in HIT [9,10,11]. It represents a challenging diagnosis and management, especially when it is associated with intracerebral hemorrhage [12,13]. The cornerstone therapy for CVS thrombosis is early anticoagulation [14]. Anticoagulants appear to be safe in patients with CVS thrombosis even if it is associated with intracranial hemorrhage [15,16,17,18]. For individuals with worsening neurological symptoms despite adequate anticoagulation endovascular thrombolysis or mechanical thrombectomy are recommended, but the limited available evidence suggests no benefit when compared with continuing anticoagulation.

We report a case of S-HIT associated with right pulmonary artery embolism, internal jugular vein, and CVS thrombosis complicated with ipsilateral acute intracerebral hemorrhage.

## 2. Case Report

**Figure 1:**
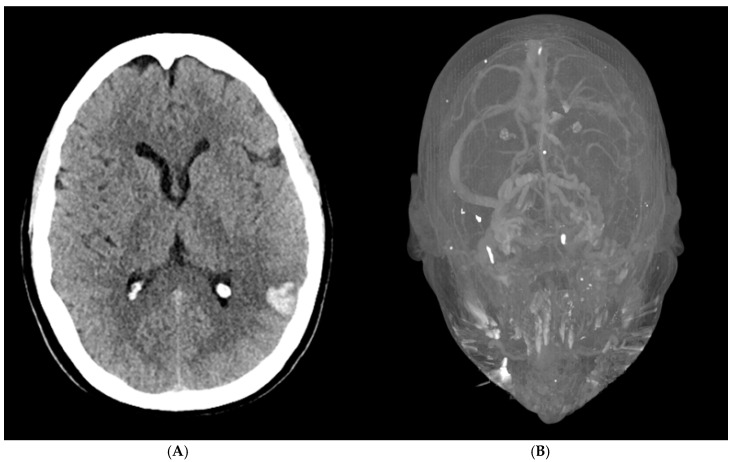
(**A**) Non-contrast CT (bottom) reveals intraparenchymal hemorrhage in the left temporoparietal region, consistent with hemorrhagic transformation due to CVST. (**B**) CT venography (top) shows cerebral venous sinus thrombosis (CVST) with thrombosis of the dural sinuses.

A 56-year-old woman presented to the emergency department with acute altered mental status, a headache localized to the left forehead, and aphasia. Her medical history was notable for severe osteoarthritis, for which she had undergone a left total knee replacement two weeks prior. Upon arrival, an initial brain CT revealed a 1.7 × 2.3 × 2.9 cm intraparenchymal hemorrhage in the posterior left temporal-parietal region, with mild surrounding vasogenic edema (Figure 1A). A follow-up CT venogram confirmed cerebral venous sinus thrombosis (CVST) involving the left transverse and sigmoid sinuses, as well as thrombosis of the upper left internal jugular vein (Figure 1B). These findings were consistent with hemorrhagic transformation in the setting of venous sinus thrombosis. A repeat brain CT the following day showed no significant interval changes, indicating a stable condition.

She was admitted to the ICU for close monitoring. On arrival, she was awake and alert, exhibiting mixed aphasia but with a Glasgow Coma Scale (GCS) of 15. She was able to follow simple axial commands such as turning her head and closing her eyes but was unable to perform a thumbs-up gesture on command. Her verbal output was present and organized, though she had trouble with word retrieval. There was no major facial asymmetry, and antigravity strength was preserved in all extremities, though the left side was difficult to assess due to her recent knee surgery. Her surgical incision appeared clean, with no signs of infection.

Initial laboratory evaluation revealed severe thrombocytopenia, with a platelet count of 43 × 10^9^/L, a significant decline from her preoperative level of 360 × 10^9^/L. Hematology was consulted prior to initiating treatment, and their recommendations included a comprehensive coagulation workup to evaluate for disseminated intravascular coagulation (DIC), thrombotic thrombocytopenic purpura (TTP), and infection-related coagulopathies, as well as PF4 antibody testing to assess for heparin-induced thrombocytopenia (HIT). Given the concern for an underlying thrombotic process and risk of worsening CVST, a multidisciplinary discussion with Neurocritical Care and Hematology led to the decision to initiate high-intensity heparin infusion. The patient was then air-transferred to a specialized Neurology ICU for further management.

Upon arrival at the Neurology ICU, her platelet count had further declined to 38 × 10^9^/L, raising concern for HIT. Heparin was promptly discontinued, and she was transitioned to a direct thrombin inhibitor, bivalirudin, for anticoagulation. Based on recommendations from Hematology, intravenous immunoglobulin (IVIG) at a dose of 1 mg/kg daily for two days was added as an additional treatment to support platelet recovery and mitigate further thrombotic complications. By hospital day 2, her platelet count began to improve.

Results from the hematologic workup revealed that DIC, TTP, and infection-related causes were effectively ruled out. However, a HIT ELISA was significantly elevated at 2.486 OD (reference < 0.400), with 100% heparin inhibition, strongly suggesting heparin-induced thrombocytopenia. Further testing showed normal ADAMTS13 activity (>100%; reference ≥ 70%), ruling out TTP. Additional laboratory findings included an extremely elevated D-dimer (>42,000 ng/mL FEU; reference ≤ 500), high LDH (352 U/L; reference 122–222), low fibrinogen (178 mg/dL; reference 200–393), and an increased reticulocyte count (4.37%; reference 0.60–2.71), all indicative of ongoing clotting and hemolysis. Autoimmune and antiphospholipid antibody testing, including beta-2 glycoprotein I (IgG and IgM), phospholipid antibodies, and lupus anticoagulant, were all negative. Protein C, protein S, and factor X levels were elevated, though the clinical significance remained unclear. Given the lack of prior heparin exposure and the constellation of thrombocytopenia, thrombosis, and strongly positive HIT markers, the findings were consistent with spontaneous heparin-induced thrombocytopenia (spontaneous HIT), a rare but serious prothrombotic disorder that mimics traditional HIT but occurs without prior heparin exposure, often triggered by alternative platelet-activating factors.

**Figure 2:**
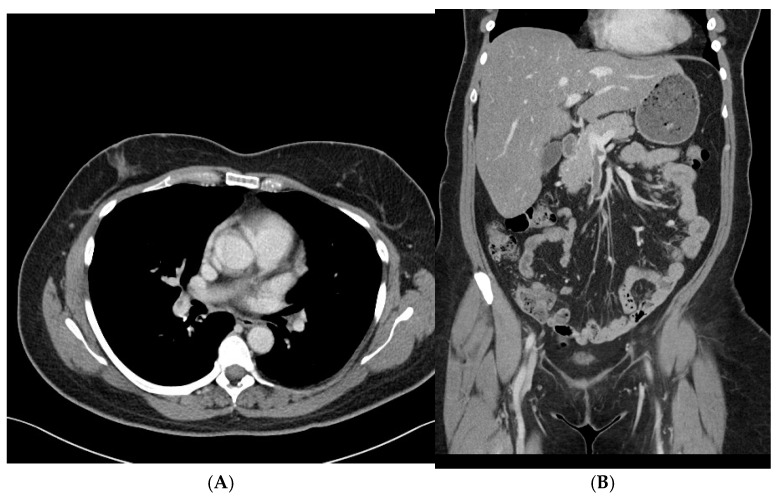
(**A**,**B**) Contrast-enhanced CT imaging shows non-occlusive thrombi in the portal and superior mesenteric veins, along with multiple non-occlusive emboli in the pulmonary arteries.

By hospital day 3, repeat brain CT and CT venogram showed that the intraparenchymal hemorrhage and venous sinus thrombosis remained stable, with no new areas of bleeding or clot extension. On hospital day 6, a CT scan of the chest, abdomen, and pelvis was obtained to evaluate for an underlying malignancy or other prothrombotic conditions. Imaging revealed a non-occlusive thrombus in the right main portal vein and superior mesenteric vein, as well as multiple non-occlusive emboli in the right superior and inferior pulmonary arteries. There was no evidence of right ventricular strain or malignancy. Given that these thrombi were identified after heparin cessation and the initiation of bivalirudin, it is possible they were already present before the transition in anticoagulation therapy, further supporting the underlying prothrombotic state associated with spontaneous HIT (Figure 2A,B).

**Figure 3:**
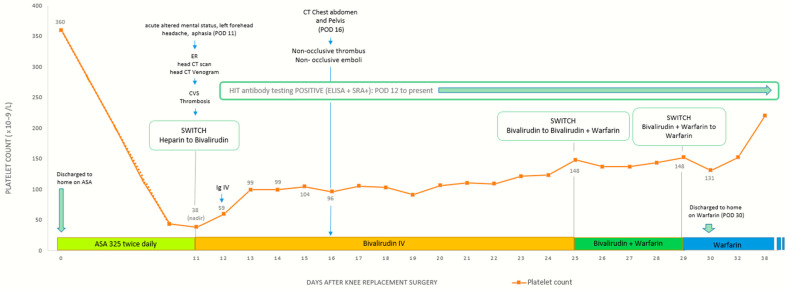
Clinical course of spontaneous HIT after knee replacement, with platelet decline, CVS thrombosis, IVIG treatment, and anticoagulation transition from heparin to bivalirudin to warfarin, leading to recovery and discharge.

Despite the extensive clot burden, her platelet count steadily improved, surpassing 150 × 10^9^/L by hospital day 12. She was subsequently transitioned to warfarin with a five-day overlap with bivalirudin to ensure adequate anticoagulation. By hospital day 17, she had stabilized and was discharged with follow-up plans, including outpatient care with family medicine, neurology, and speech therapy to aid in her recovery from aphasia (Figure 3).

## 3. Discussion

The first step in managing cerebral venous sinus (CVS) thrombosis is to start anticoagulation as early as possible, even when there is associated bleeding in the brain. The clot increases venous pressure, which can lead to hemorrhagic transformation, while also disrupting the blood–brain barrier, causing vasogenic edema and further complications. Imaging is critical for diagnosis, with CT scans detecting hemorrhagic changes and CT venography (CTV) confirming the presence of sinus occlusions, helping guide treatment decisions.

CVS thrombosis is a rare but serious complication after major orthopedic surgeries, especially total knee replacements [15]. These procedures put patients at an increased risk of blood clots due to prolonged immobility, vascular injury, and a temporary hypercoagulable state. While deep vein thrombosis (DVT) and pulmonary embolism (PE) are more commonly recognized, CVS thrombosis is a less frequent but equally important concern. To prevent blood clots, anticoagulation is routinely used after orthopedic surgery, but in cases where there is also bleeding, treatment must be carefully adjusted to prevent clot progression while minimizing the risk of further hemorrhage.

In our case, a patient who had recently undergone a left total knee replacement developed CVS thrombosis with hemorrhagic transformation and was started on high-intensity heparin. However, she soon developed severe thrombocytopenia, raising concerns for heparin-induced thrombocytopenia (HIT). Further testing confirmed spontaneous HIT, which required an immediate switch to bivalirudin for anticoagulation. Bivalirudin was chosen because of its short half-life of about 25 min, predictable anticoagulant effect, and metabolism through proteolysis rather than the liver or kidneys, making it a safer choice for a patient at risk for both blood clots and bleeding. Since its effects wear off within an hour of stopping the infusion, it provided greater control over anticoagulation, allowing treatment to be adjusted as needed.

An interesting consideration is the role of surgical trauma in triggering spontaneous HIT. Research suggests that glycosaminoglycans released from knee cartilage during orthopedic surgery could stimulate the immune system to produce anti-PF4/heparin antibodies, potentially leading to spontaneous HIT. While other factors may also contribute, this raises an important question about whether tissue damage from surgery itself could trigger HIT, even in patients who have never been exposed to heparin. This case adds to the growing evidence that HIT can develop in ways beyond traditional heparin exposure, emphasizing the importance of monitoring for unexpected thrombocytopenia in postoperative patients.

Spontaneous HIT was first recognized in 2008 when three patients were reported to have developed HIT-like symptoms without prior heparin exposure [6]. Since then, several similar cases have been documented, suggesting that spontaneous HIT is not truly spontaneous but is triggered by inflammation, tissue damage, or infection. In 2014, Warkentin et al. [15] proposed a set of diagnostic criteria for spontaneous HIT, including thrombocytopenia without an alternative explanation, thrombosis, no recent heparin exposure, strongly positive PF4-dependent enzyme immunoassays (EIAs) in at least two different assays, and a strongly positive platelet activation assay with both heparin-independent and heparin-dependent activation. Other classic HIT features, such as inhibition at high-dose heparin and Fc receptor-dependent activation, were also included in the criteria.

Our patient met all of these criteria. She had strongly positive HIT antibodies on both enzyme immunoassay (EIA) and serotonin release assay (SRA), consistent with previously reported cases. Fortunately, she did not develop arterial thrombosis, which aligns with previous HIT research showing that venous thrombosis is four times more common than arterial thrombosis. A full workup ruled out antiphospholipid syndrome, acute disseminated intravascular coagulation (DIC), thrombotic thrombocytopenic purpura (TTP), and infection, confirming that spontaneous HIT was the most likely cause.

Although a 2017 systematic review found no major differences between bivalirudin and argatroban in terms of preventing clot progression, bleeding risk, or overall mortality in HIT patients, in this case, bivalirudin proved to be an essential tool [19]. It allowed for precise control of anticoagulation in a patient dealing with both active clotting and bleeding, making it a valuable option in complex cases. This case highlights the need for personalized anticoagulation strategies in postoperative patients, particularly after major orthopedic surgery, where both thrombosis and bleeding risks must be carefully managed, especially in the setting of spontaneous HIT.

We believe our observation adds to the existing literature on S-HIT and furthers the understanding the underlying process of autoantibody formation against PF4 in the setting of post-knee replacement. This case is an example of S-HIT associated with multiple life-threatening venous thrombotic events (left transverse and sigmoid cerebral sinuses, left internal jugular, pulmonary embolism, main portal, and superior mesenteric veins) complicated with an intra-parenchymal brain bleed successfully managed with systemic anticoagulation as per recent guidelines recommendations.

## 4. Conclusions

Spontaneous heparin-induced thrombocytopenia (S-HIT), associated with cerebral venous sinus (CVS) and internal jugular (IJ) thrombosis, is a rare form of HIT that typically occurs after an inflammatory trigger, such as surgery or infection, without prior heparin exposure. In patients presenting with unexplained thrombocytopenia and thrombotic events following an inflammatory event, S-HIT should be considered as a possible diagnosis. Early recognition is crucial, as avoiding heparin exposure is the most important aspect of management, while treatment otherwise follows the standard approach for HIT. The presence of intracerebral bleeding does not necessarily preclude systemic anticoagulation in CVS thrombosis; however, careful monitoring is essential to detect any worsening hemorrhage and to assess the potential need for endovascular thrombolysis or mechanical thrombectomy.

## Data Availability

No new data were created or analyzed in this study. Data sharing is not applicable to this article as no datasets were generated or analyzed during the current study.
